# The discriminative power of patient experience surveys

**DOI:** 10.1186/1472-6963-11-332

**Published:** 2011-12-06

**Authors:** Dolf de Boer, Diana Delnoij, Jany Rademakers

**Affiliations:** 1 NIVEL (Netherlands Institute for Health Services Research), Utrecht, Netherlands; 2 CKZ (Centre for Consumer Experiences in Healthcare), Utrecht, Netherlands; 3 Scientific Centre for Transformation in Care and Welfare (Tranzo), Tilburg University, Netherlands

## Abstract

**Background:**

Comparisons of patient experiences between providers are increasingly used as an index of performance. The present study describes the ability of patient experience surveys to discriminate between healthcare providers for various patient groups and quality aspects, and reports the sample sizes required for reliable (comparisons of) provider scores.

**Method:**

The consumer quality index is a family of surveys that are tailored to specific patient groups. Data was used from patients who underwent cataract surgery, patients who underwent hip or knee surgery, patients suffering from spinal disc herniation and patients suffering from varicose veins. Multi-level regression models were fitted to assess the proportion of variance in patient experiences that is attributable to providers for various quality aspects.

**Results:**

The proportion of variance in patient experiences that is attributable to providers varied from 0.001 to 0.054. The required sample size for reliable estimates at the provider level varied from 41 to 1967 per provider. Differences in discriminative power between patient groups and/or quality aspects were inconsistent, with one exception: for all groups, the discriminative power of experiences regarding change in physical functioning was particularly limited.

**Conclusions:**

From a statistical point of view, the discriminative power appears limited. The sample sizes required for reliable estimates are often substantial and deserve careful consideration when setting up measurements. Future research should evaluate the discriminative power by validating differences between providers in patient experiences with other indices and should explore other, more sensitive measures of patient experiences regarding treatment-related changes in physical functioning.

## Background

It has been proposed that competition between healthcare providers may increase the quality and cost-effectiveness of healthcare [1]. For competition to emerge in healthcare, the availability of comparative data on the performance of health care providers is considered essential [2,3]. One way to generate such data is to measure patients' experiences of the care they received and compare those experiences between providers [4,5]. Indeed, measurement of patient experiences is now a common strategy for monitoring healthcare provider performance in a number of countries and performance information is frequently made available to facilitate consumer choice [5-12].

In the Netherlands, the Ministry of Healthcare promotes the consumer quality (CQ) index as the Dutch national standard for measuring patient experiences. The CQ-index is an instrument inspired by two other types of surveys: the American CAHPS (Consumer Assessment of Health care Providers and Systems) [4,13] and the Dutch QUOTE (QUality Of care Through the patients' Eyes) [14-17]. The CQ-index is characterized by its disease-specific and provider-specific focus as well as the assessment of patient priorities, which are both derived from QUOTE. From CAHPS, the CQ-index adopted the layout, response scales and standardized sampling, data collection, analysis and presentation. Similarly to both the CAHPS and QUOTE, the CQ-index focuses on patient experiences, rather than patient satisfaction. The underlying assumption is that measures of actual experiences with the quality of care will be less subjective than evaluative measures of satisfaction.

One of the main purposes of the CQ-index is to provide performance indicators on quality of care from the patient perspective. During the development of a CQ-index, a consortium of stakeholders is formed, which typically includes governmental bodies, associations that represent healthcare providers, health insurance companies and patient organizations [18]. This consortium is consulted and kept informed during the development of the survey to ensure the various stakeholders accept the resulting instrument and the indicators derived from the instrument. Such a consortium generally also organizes nationwide data collections for the measurement of these indicators.

In the context of institutional performance, competition and consumer information, the measurement and publication of indicators based on patient experiences is particularly informative if these indices show differences between providers. This is necessary when data is used in benchmarks in order to detect best practices and stimulate quality improvement. It is also of paramount importance if data are used as consumer information aimed at facilitating patient choice. After all, if there are no differences, there is not much to choose from. The discriminative power of the instrument must therefore be sufficient, but what is sufficient? Ideally, the discriminative power of a patient survey is at least enough to meet the following criteria: *(1) *the instrument detects significant differences between healthcare providers, and *(2) *the sample sizes required for reliable estimates at the provider level - and reliable comparison of those estimates - are available for each provider. There is of course a third criterion, namely that the differences detected between providers should reflect meaningful differences in care or service. The data available to the authors does not allow this criterion to be addressed, but this issue will be revisited in the discussion section. Naturally, criteria for the discriminative power of surveys should be met following the necessary adjustments for differences in case mix [19].

Future projects that seek to develop patient experience surveys may find empirical data that illustrates the discriminative power of such surveys in a variety of settings to be useful. Such data may guide expectations on the discriminative power of the survey under development, and may help choose the unit of analysis at which providers are compared such that the expected number of respondents required per unit of analysis may be achieved.

The means by which the discriminative power of a patient experience survey may be tested depends in part on the analytical strategy that is used. A common way to analyse data on health care provider performance that is widely recommended [19-21] and has also been adopted by the CQ-index [5,12,19] is multi-level modelling. These models resemble more common analytical strategies such as analysis of variance or regression analyses, with two important differences: *(1) *the multi-level model decomposes variance into that attributable to healthcare providers and that attributable to other sources such as individual differences, and *(2) *the multi-level model accounts for the fact that individuals within healthcare providers are not independent from one another [19,22]. As a general assessment of differences between providers, the variance attributable to providers can be tested for significance. The magnitude of the variance between healthcare providers may then be expressed as a proportion of the total variance on a scale from 0 to 1 (intra-class correlation coefficient; ICC). Additionally, comparisons between healthcare providers can be made to determine whether a given healthcare provider differs significantly from any of the other healthcare providers.

Several studies have reported the ICC's for patient experience surveys. For example, Stubbe et al. reported that the ICC's for cataract surgery varied from not significant (nurses communication) to .03 (ophthalmologist's communication) [12]. In another study, the ICC's for hip or knee surgery were reported to vary from not significant (communication about medication, pain control, global rating of hospital) to .03 (doctor's communication, nurse's communication) [5]. Furthermore, Damman et al. reported that the ICC's for health plans varied from .02 (health plan information) to .05 (global rating) [19]. In addition, Zaslavsky et al [23] reported the percentage of variance in experiences that was explained by health plan and a number of geographical variables. For the vast majority of quality aspects, the variance explained varied from 0.4% to 6.0%, which corresponds to the ICC's reported in the aforementioned studies. Further, Hargraves et al. [4] reported the number of respondents required for reliable estimates of performance scores per health plan, which is also indicative of the magnitude of differences between providers, as fewer observations are required when differences are large. For global ratings, the required number of respondents varied from 49 (global rating of health plan) to 287 (global rating of specialist). For composite measures, the required number of respondents varied from 64 (getting the care that was needed) to 169 (doctors who communicate). Although it was concluded that the plan-level reliability was impressive, it is also worth noting that with response rates varying from 24 percent to 57 percent between plans, sample sizes should exceed 500 for most plans to obtain the required number of respondents for reliable estimates at the provider level for both the global ratings and the composite measures. Solomon et al., [24] reported on a survey to evaluate the performance of medical groups. The required sample size for reliable scores at the medical group level was reported to vary from 52 (access to care) to 1340 (preventive counselling). Finally, Keller et al., [25] also reported the reliability of performance scores of composite measures at the hospital level. They assumed a response of 300 per hospital and most reliabilities appeared satisfactory, ranging from 0.66 (medicine communication) to 0.89 (nurse communication; responsiveness). To sum up, although studies do point in broadly the same direction, there are differences between studies regarding ICC's or regarding the required number of respondents for a satisfactory unit-level reliability. As such, it is intriguing what drives these differences.

The Consumer Quality Index (CQ-index) is a family of surveys for measuring the patient perspective that allows us to examine the magnitude and reliability of differences between health care providers in various patient groups for various quality aspects. In the present study, we seek to describe the discriminative power of CQI surveys for several quality aspects in various settings. Data was used from patients suffering from varicose veins, patients who underwent hip or knee surgery, patients who underwent cataract surgery and patients suffering from spinal disc herniation.

The following research questions will be addressed:

1. What is the discriminative power of the patient surveys at issue?

2. Does the discriminative power of patient surveys vary across different measures and/or patient groups?

3. What sample sizes are required for reliable estimates of provider scores?

## Methods

### Participants

All data was collected in the Netherlands using self-administered surveys. Patients were identified through insurance companies and/or hospitals and approached by mail on up to four occasions: an initial questionnaire accompanied by a letter, a thank you/reminder note one week later, a reminder mailing for non-respondents that consisted of the questionnaire and a letter another three weeks later and a final reminder letter for non-respondents another two weeks later. The dataset for patients who underwent hip or knee surgery consisted of 1514 patients from 43 hospitals (response = 75.0%), the dataset for patients who underwent cataract surgery consisted of 4126 patients from 55 hospitals (response = 71.7%), the dataset for varicose veins consisted of 2195 participants from 20 hospitals (response = 61.5%) and the dataset for spinal disc herniation contained 1648 patients from 20 hospitals (response = 42.3%). The number of observations per provider varies within and between the datasets used, but since the present paper does not report estimates for individual providers this presents no major limitation. Data on the demographic characteristics (age, self-observed health, education and gender) is presented in Table 1.

**Table 1:** Demographic characteristics of the patient populations

	Age						Education						General health										Gender			
	18-44		45-64		65+		Low		Medium		High		Poor		Fair		Good		Very good		Excellent		Male		Female	
Hip or knee surgery	29	2%	413	27%	1072	71%	962	64%	397	26%	155	10%	12	1%	196	13%	785	52%	260	17%	261	17%	432	29%	1082	71%
Varicose veins	640	29%	1212	55%	343	16%	645	29%	948	43%	602	27%	19	1%	278	13%	1371	62%	362	16%	165	8%	394	18%	1801	82%
Cataract surgery	34	1%	628	16%	3374	84%	2551	63%	1009	25%	476	12%	96	2%	1143	28%	2045	51%	473	12%	279	7%	1522	38%	2514	62%
Spinal disc herniation	493	30%	826	50%	329	20%	562	34%	667	40%	419	25%	69	4%	504	31%	818	50%	184	11%	73	4%	846	51%	802	49%

The studies in which the data was collected were performed in accordance with the Declaration of Helsinki. Research by means of surveys that are not taxing and/or hazardous for patients is not subject to the Dutch Medical Research Involving Human Subjects Act (WMO). Accordingly, ethical approval was not required. All surveys were accompanied by instructions including a statement that participation is voluntarily and anonymous.

### Selection of patient experiences

For the purposes of the present study, we selected experiences with patient-doctor communication and experiences regarding the effect of treatment in terms of changes in physical functioning as, for these experiences, composite measures could be calculated for each survey. The items underlying composite scores for patient-doctor communication are presented in Table 2, along with their internal consistency (Cronbach's coefficient alpha: 0.81 - 0.92). The response categories for these items were: never-sometimes-usually-always. The items vary somewhat between surveys, as surveys are developed in separate projects, each with a separate consortium of stakeholders that is consulted for decisions on the content of questionnaires. Furthermore, composite scores were calculated for the extent to which relevant elements of physical functioning were improved as compared to the start of treatment. For all surveys, the response categories regarding physical functioning were "worse-similar-better" than before treatment. For the survey for patients that underwent a cataract surgery, items underlying this composite score contained 12 items (Cronbach's coefficient alpha = 0.90) covering issues such as being able to see things from a close distance or far away, being able to cope with bright lights, being able to drive etc. For the survey on hip or knee surgery, the composite score also consisted of 12 items (Cronbach's coefficient alpha = 0.95) and covered issues such as stair climbing, pain, standing, walking etc. In the case of varicose veins, this composite entailed 9 items (Cronbach's coefficient alpha = 0.91) and covered issues such as feelings of fatigue in the legs, pain, standing, physical appearance etc. For spinal disc herniation, the composite contained 22 items (Cronbach's coefficient alpha = 0.94) and covered issues such as stair climbing, standing up, walking, back pain, mobility etc. Finally, each survey contained a global rating of care and a question addressing the extent to which a patient would recommend his or her healthcare provider to family and friends; both were included in the analyses for the present paper.

**Table 2:** The items that underlie the composite doctor's communication for the various patient groups

	Varicose veins (α = 0.90)	Cataract (α = 0.82)	Hip- or knee surgery (α = 0.81)	Rheumatoid arthritis (α = 0.82)	Spinal disc herniation (α = 0.92)
Doctor takes me seriously	x		x		
Doctor listens attentively	x	x	x	x	x
Doctor takes enough time	x	x	x	x	
Doctors treat me with respect and dignity	x	x	x		
Doctor explains clearly	x		x	x	x
Being able to ask questions				x	x
Getting clear answers	x				x

### Data analyses

The discriminative power of the surveys at issue was assessed using multi-level modelling. For all surveys, the models included two levels: the individual and the healthcare provider. The healthcare provider is the hospital or hospital department rather than an individual doctor, as reporting quality scores for individual doctors is a heavily debated issue in the Netherlands with regard to privacy legislation. In addition, it is unlikely that healthcare providers would cooperate with quality measurements if results would be reported per individual doctor.

We first fitted a series of empty models and calculated the intra-class correlation coefficient (ICC). The ICC reflects the proportion of total variance that is attributed to between-provider differences and is used as a general measure of discriminatory power. Subsequently, we accounted for the variables age, education and self-rated health, which are commonly identified as case mix adjusters and evaluated the impact of this case mix adjustment by its effect on the ICC. In the case of experienced change in physical functioning, self-rated health was not included in the case-mix-adjusted model as it is plausible that patients who experience no change or worsening of their physical functioning would also rate their own health as lower compared to patients whose physical functioning improved. Accordingly, adjustment for self-rated health would remove real differences in experienced change in physical functioning. Further, the range in which 95% of the providers' means are expected to occur was determined as the average across all provider means plus or minus two standard deviations (SD), where the SD is calculated as the square root of the variance at the provider level. The required number of respondents to achieve a reliability at the provider level of 0.70 or 0.80 was also calculated [[22], p59]. In contrast to the reliability indicated by Cronbach's coefficient alpha - where items of the same composite are expected to agree within individuals as they measure the same construct - the provider level reliability is based on the theory that patients treated by the same provider should agree in their assessments of that provider. If agreement between patients from the same provider is limited, more respondents are required to achieve a reliable estimate of the performance of that provider.

## Results

The ICC's for the empty and the corrected models are presented in Table 3. As can be seen in Table 3, the corrected models generally display a reduced ICC compared to the empty models, suggesting that some of the differences between healthcare providers that are observed in the empty model may be explained by differences in their populations on the case mix adjusters. This phenomenon was least pronounced for the global rating (see Table 3).

**Table 3:** The discriminative power of patient experience surveys for different patient groups and quality aspects in unadjusted and adjusted models, accompanied by the sample sizes required to detect differences between providers reliably.

	Empty model			Adjusted model											
		
									95% expected range of provider scores			Required sample size per provider		Required number of patients to be approached per provider^c^	
									
	Variance providers^a^	Variance individuals^b^	ICC	Variance providers^a^	Variance individuals^b^	ICC	Mean	SD providers	lower limit	upper limit	range	reliability = .70	reliability = .80	reliability = .70	reliability = .80
*Doctor's communication*															
Hip or knee surgery (n1 = 43; n2 = 1462)^c^	**0.0133**	0.2955	0.043	**0.0100**	0.2818	0.034	3.44	0.10	3.24	3.64	0.40	65	112	87	150
Varicose veins (n1 = 20; n2 = 2189)	**0.0049**	0.2118	0.023	**0.0041**	0.2040	0.020	3.62	0.06	3.49	3.75	0.26	117	200	190	325
Cataract surgery (n1 = 55; n2 = 4021)	**0.0035**	0.1943	0.017	**0.0033**	0.1913	0.017	3.58	0.06	3.46	3.70	0.23	134	230	187	321
Spinal disc herniation (n1 = 20; n2 = 1574)	0.0025	0.4125	0.006	0.0015	0.4020	0.004	3.52	0.04	3.44	3.60	0.16	614	1053	1452	2489
*Change in physical functioning*															
Hip or knee surgery (n1 = 43; n2 = 1345)	0.0046	0.3168	0.014	0.0038	0.3105	0.012	2.50	0.06	2.38	2.63	0.24	193	331	258	442
Varicose veins (n1 = 20; n2 = 1663)	**0.0047**	0.2117	0.022	**0.0044**	0.2100	0.020	2.46	0.07	2.32	2.59	0.26	112	192	181	310
Cataract surgery (n1 = 55; n2 = 2982)	0.0003	0.1675	0.002	0.0002	0.1653	0.001	2.56	0.01	2.54	2.59	0.05	2516	4313	3494	5990
Spinal disc herniation (n1 = 20; n2 = 1592)	0.0022	0.2720	0.008	0.0010	0.2560	0.004	2.43	0.03	2.37	2.49	0.13	569	975	1355	2322
*Global rating *															
Hip or knee surgery (n1 = 43; n2 = 1496)	0.0332	1.8371	0.018	0.0215	1.7036	0.012	8.36	0.15	8.07	8.65	0.59	185	317	247	423
Varicose veins (n1 = 20; n2 = 2169)	**0.0952**	1.7139	0.053	**0.0915**	1.6076	0.054	7.92	0.30	7.32	8.52	1.21	41	70	67	114
Cataract surgery (n1 = 55; n2 = 3967)	**0.0417**	1.7341	0.023	**0.0396**	1.6791	0.023	7.75	0.20	7.35	8.15	0.80	99	169	138	236
Spinal disc herniation (n1 = 20; n2 = 1590)	0.0486	2.4523	0.019	0.0450	2.3155	0.019	7.42	0.21	7.00	7.84	0.85	120	206	284	487
*Recommendation to others*															
Hip or knee surgery (n1 = 43; n2 = 1497)	0.0076	0.3556	0.021	0.0063	0.3424	0.018	3.58	0.08	3.42	3.74	0.32	126	217	168	289
Varicose veins (n1 = 20; n2 = 2154)	**0.0182**	0.3900	0.045	**0.0170**	0.3738	0.043	3.34	0.13	3.08	3.60	0.52	51	88	84	143
Cataract surgery (n1 = 55; n2 = 4003)	**0.0111**	0.3055	0.035	**0.0108**	0.3008	0.035	3.41	0.10	3.20	3.62	0.41	65	112	91	156
Spinal disc herniation (n1 = 20; n2 = 1564)	**0.0160**	0.5157	0.030	**0.0146**	0.4986	0.028	3.16	0.12	2.92	3.40	0.48	80	137	188	323

^a ^Variances in bold are significant (p < .05)

^b ^The significance of variances at the level of individuals is not reported

^c ^Derived from the required sample size and the response rate (hip or knee surgery (75%), varicose veins (62%), cataract surgery (72%), spinal disc herniation (42%))

^d ^n1 denotes the number of healthcare providers, n2 denotes the total number of patients

Focussing on the adjusted model - which is arguably the model of choice [26,27] - it can be observed that the ICC varies from 0.001 (change in physical functioning; cataract surgery) to 0.054 (global rating; varicose veins). In a number of cases, the variance at the level of the healthcare provider was not statistically significant. This was particularly the case for change in physical functioning: the variance at the level of healthcare providers was significant only for varicose veins. Further, variances at the level of healthcare providers were not significant for doctors' communication in spinal disc herniation, the global rating for both spinal disc herniation and hip or knee surgery and recommendation to others for hip or knee surgery (see Table 3). In sum, the extent to which differences in experiences between individuals are attributable to their healthcare providers appears limited and the variance observed at the level of healthcare providers is often not significant.

To further examine the extent to which the surveys at issue are able to distinguish between health care providers, we also calculated the range in which 95% of the provider means are expected to occur, given the variance at the level of healthcare providers (see Table 3). For the two variables that consisted of items containing four response categories, the range varied from 0.16 to 0.40 (doctor's communication) and 0.32 to 0.52 (recommendation to others). For the global rating, the range varied from 0.59 to 1.21 points and for changes in physical functioning, which consisted of items containing three response categories, the expected range varied from 0.05 to 0.26. It is worth noting that, although the global rating was the measure that discriminated best between providers only for varicose veins, the expected range of provider means was the largest across all patient groups.

In addition, the number of observations per provider for reliable estimates of healthcare provider scores and, accordingly, meaningful comparison of provider scores, was calculated for a reliability of 0.70 and a reliability of 0.80. Subsequently, the number of participants that should be approached to achieve the required number of observations, given the observed response rate was assessed. In cases where the discriminative power was small (ICC < 0.01), required sample sizes per healthcare provider were large (569 - 2516) for a reliability of 0.70 and excessive (975 - 4313) for a reliability of 0.80. For the other measures, the required sample size varied from 41 to 193 for a reliability of 0.70 and from 70 to 331 for a reliability of 0.80 (see Table 3). The number of participants that should be approached for reliable estimates at the provider level is dictated by the required sample size and the expected response rate. In the last two columns of Table 3, the number of patients that should be approached is presented, again for a reliability of 0.70 and a reliability of 0.80. Obviously, the number of patients that should be approached is higher than the required sample size in all cases. The magnitude of the difference between the two is determined by the response rate: in case of spinal disc herniation (response rate = 42%), the number of patients that should be approached is more than twice the required sample size whereas in case of hip or knee surgery (response rate = 75%) the number of respondents is only about 1/3 higher (see Table 3).

## Discussion

The present study showed that the extent to which patient experiences are dependent on differences between providers is limited. The extent to which patient experiences are determined by provider differences varied from 0.001 to 0.054, which means that 0.1% to 5.4% of the variance in patient experiences may be attributed to health care providers. Accounting for common case mix adjusters generally reduced the extent to which patient experiences are attributable to providers. Further, differences in discriminative power between patient groups and/or measures were inconsistent, with one exception: for all patient groups the discriminative power of experiences regarding change in physical functioning was particularly limited. As expected, the required number of patients to approach per provider was exceptionally large in cases where the discriminative power of a measure was low and response rates were low.

The discriminative power of the various patient experience surveys as presented here is largely consistent with previous reports [4,5,12,19]. However, where it may be difficult to evaluate the parallels between previous reports, as the experiences reported varied and the methodology used was not always consistent, the present study provides a comprehensive overview for different patient groups using corresponding measures for patient experiences and identical methods for data analyses.

Whether the reported levels of discriminative power should be considered meaningful remains a matter of debate. It may be argued that the extent to which patient experiences are attributable to healthcare providers is low and that the range in which 95% of provider scores are expected to occur is rather narrow. On the other hand, empirical data on the discriminative power of a wide variety of measures in primary care - including measures such as the short form 36 and the hospital anxiety and depression score, as well as blood pressure and cholesterol - showed that the median ICC is 0.01 when looking at models without covariates and 0.005 for models including covariates [28]. These values are exceeded by most of the measures of patient experiences presented in the present paper. It may be questioned however, whether the discriminative power can be evaluated by statistical parameters alone. Ideally, the differences between providers revealed by patient experience surveys should be considered in the context of data on other measures on the same quality aspects that are independent of patient experiences. When evaluating the discriminative power of patient experience surveys regarding doctor's communication for example, it would be helpful to know how independent observers would rate the communication skills of a doctor at the lower versus the higher end of the range. Such information would illustrate the meaning of differences in patient experiences between providers.

The discriminative power of patient experiences varied between measures and surveys. One consistent trend that was observed was the limited discriminative power of experiences regarding changes in relevant elements of physical functioning following treatment. Admittedly, the development of such measures as indices of healthcare provider performance is far from complete. Accordingly, it is possible that providers do differ in terms of the experienced change in physical functioning, but that the retrospective measures used to assess these differences in the present paper are not sufficiently sensitive. In this context, it should be acknowledged that measures of changes in physical functioning have been successfully used to compare the effects of various healthcare interventions, albeit in a different format using pre- and post measurements [29,30]. Such a strategy would also allow a more advanced case-mix adjustment as the pre measurement may be used to account for differences in baseline health status. However, the use of pre- and post measurements in the context of continuous nationwide monitoring of patient experiences would substantially increase costs and respondent burden. Therefore, the CQ-index initially attempted to incorporate assessment of experiences regarding change in physical functioning, in a single measurement. Nevertheless, since the present strategy failed to demonstrate differences between providers, future attempts to adopt measures of experienced change in physical functioning as indices of provider performance should consider alternative strategies including those containing pre- and post measurements [30].

The present paper also reported the required number of patients to be approached for reliable estimates at the provider level, and accordingly for meaningful comparison of provider scores. The number of patients that should be approached is dependent on two things: the discriminative power of the survey and the response rate. The present paper showed that the number of patients to be approached is often well in excess of 100, and may even reach thousands should a comparison be desired between providers for measures of patient experiences where these differences between providers are small. In our experience, the number of patients to be approached per provider is a heavily debated issue among researchers and stakeholders when setting up measurements. On the one hand, it is appealing to keep down the number of patients to be approached to reduce costs and to prevent exclusion of small providers. On the other hand, larger numbers of patients allow more reliable estimates of provider scores and permit more and better distinctions between providers. For measures where the required number of patients to be approached for reliable estimates at the provider level is excessive due to a lack of differences between providers, we recommend that stakeholders consider whether such measures are useful for benchmarking purposes. It is unlikely of course that a benchmark would distinguish between providers in such cases, but on occasion it may be useful to illustrate that for some elements of care it does not matter which provider is chosen.

Practical dilemmas arise when the number of patients to be approached for reliable estimates of provider scores is not excessive in itself, but can still not be achieved by most providers e.g. because the type of care at issue is delivered by small providers that only treat a limited number of patients a year. In such cases, strategies to increase the number of patients that can be approached per healthcare provider are of interest. For example, where results normally reflect patient experiences in the preceding year to ensure recent and up-to-date figures, this period may be lengthened. In addition, small providers are sometimes part of a larger organization. If there is sufficient uniformity of care provision within this organization, it may be possible to choose the unit of analysis at the level of the organization, rather than at the level of the providers underlying the organization.

It should be noted that increasing the number of patients to be approached does not resolve issues of generalisability of results in case of a low response rate. Nonetheless, on the assumption that causes for non-response are broadly similar between providers and/or that possible response bias may be addressed through case mix adjustment, it may still be interesting to compare the experiences from respondents between providers. In this context it may be useful to adjust the number of patients to be approached such that there will be sufficient observations for comparing providers.

Several limitations deserve consideration when interpreting the present findings. First, the variance at the level of providers, would partially depend on the heterogeneity of the sample of providers. A more heterogeneous sample of providers would result in a larger variance on the level of providers, an increased ICC and a reduced number of patients to be approached. Whether the heterogeneity of the sample of providers is representative of the heterogeneity of all providers is difficult to determine. In addition, the heterogeneity of providers may vary between countries and/or health care systems. Nonetheless, it should be noted that the ICC's reported in the present article are broadly similar to those reported elsewhere [5,12,19,23], suggesting that if the accuracy of the observed variances could be improved, it is unlikely that this would lead to fundamentally different results. Second, it is possible that the variance at the level of individuals is under or overestimated as a result of measurement error, which is an often ignored source of variance. Accordingly, it remains essential to develop surveys that are reliable, valid and sensitive. Third, the level of the health care provider consisted of hospitals rather than individual doctors or nurses since reporting quality scores on individual health care providing staff is still a matter of debate in the Netherlands. Nevertheless, it is possible that differences between individual doctors or nurses are larger than differences between hospitals or hospital departments as assessing differences between individual nurses or doctors presents a more specific measurement. Indeed, evidence on patient reports of individual doctors showed a wider range of ICC's, varying from 0.02 to 0.17 [31]. Thus, although reporting quality scores on individual doctors appears a sensitive issue, it is certainly appealing from a methodological point of view.

## Conclusions

In conclusion, the discriminative power of patient experience surveys remains an important issue in the development of indices of healthcare provider performance. The present paper showed that the discriminative power of patient experience surveys is generally limited, but for most patient groups several measures provided sufficient discriminative power to allow reliable estimates of provider scores and, accordingly, meaningful comparisons of provider scores using sample sizes that can be achieved by most providers. In particular, differences between providers were small for items focusing on changes in physical functioning as indices of healthcare provider performance. Future research should explore other strategies for measuring patient experiences regarding change in physical functioning, intending to identify more sensitive measurement strategies. Other studies and projects may also benefit from overviews such as those given in the present paper when setting up data collection and determining the level of aggregation at which comparisons between healthcare providers are performed.

## Competing interests

The authors declare that they have no competing interests.

## Authors' contributions

DdB conceived the study, performed analyses and drafted the manuscript. DD and JR were involved in the interpretation of findings and drafting the manuscript. All authors read and approved the final manuscript.

## Pre-publication history

The pre-publication history for this paper can be accessed here:

http://www.biomedcentral.com/1472-6963/11/332/prepub

## References

[B1] Porter ME Teisberg EO Redefining Health Care 2006 Harvard Business School Publishing

[B2] Fung CH Lim YW Mattke S Damberg C Shekelle PG Systematic review: the evidence that publishing patient care performance data improves quality of care Ann Intern Med 2008 148 111 123 18195336 10.7326/0003-4819-148-2-200801150-00006

[B3] Porter ME Teisberg EO Redefining competition in health care Harv Bus Rev 2004 82 64 76 136 15202288

[B4] Hargraves JL Hays RD Cleary PD Psychometric properties of the Consumer Assessment of Health Plans Study (CAHPS) 2.0 adult core survey Health Serv Res 2003 38 1509 1527 10.1111/j.1475-6773.2003.00190.x 14727785 PMC1360961

[B5] Stubbe JH Gelsema T Delnoij DM The Consumer Quality Index Hip Knee Questionnaire measuring patients' experiences with quality of care after a total hip or knee arthroplasty BMC Health Serv Res 2007 7 60 10.1186/1472-6963-7-60 17462084 PMC1876799

[B6] Fritt Sykehusvalg http://www.frittsykehusvalg.no/start/

[B7] Your Hospitals http://www.health.vic.gov.au/yourhospitals/

[B8] Østerbye T Sevaldsen J Skjødt Hansen K Freil M Patient's experiences in Danish hospitals 2007 http://www.patientoplevelser.dk/log/medie/Rapporter/Survey_2006_english.pdf22148131

[B9] CAHPS Home Page https://www.cahps.ahrq.gov/default.asp

[B10] Redding D Boyd J Data briefing. Involvement leads to satisfaction Health Serv J 2008 17 19018642

[B11] Delnoij DMJ Asbroek GT Arah O Koning JD Poll A Vriens B Schmidt P Klazinga NS Made in the USA: the import of American Consumer Assessment of Health Plan Surveys (CAPHS) into the Dutch social insurance system European Journal of Public Health 2006 16 652 659 10.1093/eurpub/ckl023 16524940

[B12] Stubbe JH Brouwer W Delnoij DM Patients' experiences with quality of hospital care: the Consumer Quality Index Cataract Questionnaire BMC Ophthalmol 2007 7 14 10.1186/1471-2415-7-14 17877840 PMC2093924

[B13] Carman KL Short PF Farley DO Schnaier JA Elliott DB Gallagher PM Epilogue: Early lessons from CAHPS Demonstrations and Evaluations. Consumer Assessment of Health Plans Study Med Care 1999 37 MS97 105 10.1097/00005650-199903001-00011 10098564

[B14] Hekkink CF Sixma HJ Wigersma L Yzermans CJ Van Der Meer JT Bindels PJ Brinkman K Danner SA QUOTE-HIV: an instrument for assessing quality of HIV care from the patients' perspective Qual Saf Health Care 2003 12 188 193 10.1136/qhc.12.3.188 12792008 PMC1743708

[B15] Nijkamp MD Sixma HJ Afman H Hiddema F Koopmans SA van den Borne B Hendrikse F Nuijts RM Quality of care from the perspective of the cataract patient. QUOTE cataract questionnaire J Cataract Refract Surg 2002 28 1924 1931 10.1016/S0886-3350(02)01373-1 12457664

[B16] Sixma HJ Kerssens JJ Campen CV Peters L Quality of care from the patients' perspective: from theoretical concept to a new measuring instrument Health Expect 1998 1 82 95 10.1046/j.1369-6513.1998.00004.x 11281863 PMC5139902

[B17] van Campen C Sixma HJ Kerssens JJ Peters L Rasker JJ Assessing patients' priorities and perceptions of the quality of health care: the development of the QUOTE-Rheumatic-Patients instrument Br J Rheumatol 1998 37 362 368 10.1093/rheumatology/37.4.362 9619883

[B18] Delnoij DM Rademakers JJ Groenewegen PP The Dutch consumer quality index: an example of stakeholder involvement in indicator development BMC Health Serv Res 2010 10 88 10.1186/1472-6963-10-88 20370925 PMC2864255

[B19] Damman OC Stubbe JH Hendriks M Arah OA Spreeuwenberg P Delnoij DM Groenewegen PP Using multilevel modeling to assess case-mix adjusters in consumer experience surveys in health care Med Care 2009 47 496 503 10.1097/MLR.0b013e31818afa05 19238105

[B20] Arling G Lewis T Kane RL Mueller C Flood S Improving quality assessment through multilevel modeling: the case of nursing home compare Health Serv Res 2007 42 1177 1199 10.1111/j.1475-6773.2006.00647.x 17489909 PMC1955250

[B21] Goldstein H Spiegelhalter DJ League Tables and their Limitations: Statistical Issues in Comparisons of Institutional Performance Journal of the Royal Statistcial Society Seris A (Statistics in Society) 1996 159 385 443 10.2307/2983325

[B22] Snijders TAB Bosker RJ Multilevel Analysis: An introduction to basic and advanced multilevel modeling 1999 Sage Publishers

[B23] Zaslavsky AM Zaborski LB Cleary PD Plan, geographical, and temporal variation of consumer assessments of ambulatory health care Health Serv Res 2004 39 1467 1485 10.1111/j.1475-6773.2004.00299.x 15333118 PMC1361079

[B24] Solomon LS Hays RD Zaslavsky AM Ding L Cleary PD Psychometric properties of a group-level Consumer Assessment of Health Plans Study (CAHPS) instrument Med Care 2005 43 53 60 15626934

[B25] Keller S O'Malley AJ Hays RD Matthew RA Zaslavsky AM Hepner KA Cleary PD Methods used to streamline the CAHPS Hospital Survey Health Serv Res 2005 40 2057 2077 10.1111/j.1475-6773.2005.00478.x 16316438 PMC1361248

[B26] O'Malley AJ Zaslavsky AM Elliott MN Zaborski L Cleary PD Case-mix adjustment of the CAHPS Hospital Survey Health Serv Res 2005 40 2162 2181 10.1111/j.1475-6773.2005.00470.x 16316443 PMC1361241

[B27] Zaslavsky AM Statistical issues in reporting quality data: small samples and casemix variation Int J Qual Health Care 2001 13 481 488 10.1093/intqhc/13.6.481 11769751

[B28] Adams G Gulliford MC Ukoumunne OC Eldridge S Chinn S Campbell MJ Patterns of intra-cluster correlation from primary care research to inform study design and analysis J Clin Epidemiol 2004 57 785 794 10.1016/j.jclinepi.2003.12.013 15485730

[B29] Moser DK Yamokoski L Sun JL Conway GA Hartman KA Graziano JA Improvement in health-related quality of life after hospitalization predicts event-free survival in patients with advanced heart failure J Card Fail 2009 15 763 769 10.1016/j.cardfail.2009.05.003 19879462 PMC2772831

[B30] Browne J Jamieson L Lewsey J van der Meulen J Black N Cairns J Lamping D Smith S Copley L Horrocks H Patient Reported Outcome Measures (PROMs) in Elective Surgery 2007 http://www.lshtm.ac.uk/php/hsrp/research/proms_report_12_dec_07.pdf

[B31] Hays RD Chong K Brown J Spritzer KL Horne K Patient reports and ratings of individual physicians: an evaluation of the DoctorGuide and Consumer Assessment of Health Plans Study provider-level surveys Am J Med Qual 2003 18 190 196 10.1177/106286060301800503 14604271

